# How do we define high and low dose intensity of heart failure medications: a scoping review

**DOI:** 10.1186/s12872-023-03514-2

**Published:** 2023-09-27

**Authors:** Min Ji Kwak, Qian Wang, Chukwuma Onyebeke, Travis Holder, Parag Goyal, Rajender Aparasu, Abhjeet Dhoble, Holly M. Holmes

**Affiliations:** 1 The University of Texas Health Science Center at Houston, 6431 Fannin Street, Houston, TX 77030 USA; 2 Department of Medicine, Weill Cornell Medicine, New York, NY USA; 3 Texas Medical Center Library, Houston Academy of Medicine, Texas Medical Center, Houston, TX USA; 4 Department of Pharmaceutical Health Outcomes and Policy, University of Houston, Houston, TX USA

**Keywords:** Heart failure, Pharmacotherapy, Scoping review

## Abstract

**Background:**

Older adults with heart failure often experience adverse drug events with high doses of heart failure medications. Recognizing whether a patient is on a high or low dose intensity heart failure medication can be helpful for daily practice, since it could potentially guide the physician on which symptoms to look for, whether from overdosing or underdosing. However, the current guideline does not provide sufficient information about the dose intensity below the target dose. Furthermore, the definition of high or low-intensity heart failure medication is unclear, and there is no consensus.

**Methods:**

To close the knowledge gap, we conducted a scoping review of the current literature to identify the most frequently used definition of high versus low doses of heart failure medications. We searched Pubmed, Embase, CINAHL, and Cochrane Library using comprehensive search terms that can capture the intensity of heart failure medications.

**Results:**

We reviewed 464 articles, including 144 articles that had information about beta-blockers (BB), 179 articles about angiotensin-converting enzyme inhibitors (ACEi), 75 articles about angiotensin receptor blockers (ARB), 80 articles about diuretics, 37 articles about mineralocorticoid receptor antagonists (MRA), and 33 articles about angiotensin receptor-neprilysin inhibitor (ARNI). For hydralazine with isosorbide dinitrate or ivabradine, we could not identify any eligible articles. We identified 40 medications with most frequently used definitions of dose intensity. Four medications (nadolol, pindolol, cilazapril, and torsemide) did not reach consensus in definitions. Most of the BBs, ACEis, or ARBs used the definition of low being < 50% of the target dose and high being ≥ 50% of the target dose from the guideline. However, for lisinopril and losartan, the most commonly used definitions of high or low were from pivotal clinical trials with a pre-defined definition of high or low.

**Conclusion:**

Our comprehensive scoping review studies identified the most frequently used definition of dose intensity for 40 medications but could not identify the definitions for 4 medications. The results of the current scoping review will be helpful for clinicians to have awareness whether the currently prescribed dose is considered high - requiring close monitoring of side effects, or low - requiring more aggressive up-titration.

**Supplementary Information:**

The online version contains supplementary material available at 10.1186/s12872-023-03514-2.

## Background

Over the last decade, the management of heart failure, especially heart failure with reduced ejection fraction, has evolved as a result of multiple randomized clinical trials that have identified several medications that improve the survival of patients with heart failure [1, 2]. Therefore, the clinical practice guidelines for the management of heart failure recommend multiple medications for optimizing care of adults with heart failure, along with specified doses that are recommended to target [3]. Target doses are based on findings from clinical trials showing increased clinical benefits at higher doses. However, there is a gap between target doses and prescribed doses in the real world; a relatively small fraction of patients with heart failure receive target doses in clinical practice [4–6].

Factors that may be associated with suboptimal prescription includes older age, lower systolic blood pressure, or renal insufficiency. Furthermore, patients with heart failure, especially older adults, tend to have other medical conditions requiring significant numbers of medications, leading to polypharmacy (taking 5 or more medications) and adverse drug events [7–9]. Therefore, finding the balance between prescribing the appropriate dose and number of heart failure medications to achieve clinical benefit and avoiding adverse drug events is crucial in managing heart failure among older adults. However, the current guideline promoting the target dose of each medication does not provide sufficient information about the effect of other dosing options below the target doses or about the utility of certain combinations of lower or higher doses of different medications. Currently, there are no established definitions of high or low doses of heart failure medications. Therefore, clinicians prescribing heart failure medications do not have much insight if the currently prescribed dose is considered high, requiring close monitoring of side effects, or the dose is relatively low, requiring more aggressive up-titration. To close this knowledge gap, we conducted a scoping review to identify the most frequently used definitions of high versus low doses of heart failure medications in the current literature.

## Methods

### Scoping review

We chose to conduct a scoping review over a systematic review since the article’s purpose is to map the evidence associated with the definitions of high and low doses of heart failure medications and attempt to clarify the definition, which fits the overall objective of scoping review [10]. We followed the PRISMA-ScR checklist (supplemental file 1) in developing the protocol and methodology [11]. Questions to answer through this review were: In the research regarding heart failure medications, (1) What is the most frequently used definition of high and low dose in heart failure medications, (2) How often did was a rationale provided for the definition, and (3) What type of research was conducted?

### Search strategy

The electronic databases Ovid Medline, Elsevier Embase, EBSCO CINAHL, and Cochrane Library were searched by a research librarian (TH) using comprehensive search strategies that can capture the intensity of heart failure medications (search terms available on supplemental file 2). We conducted the search on 12/18/2020. Prior protocol registration was not done for this scoping review.

### Inclusion criteria

Population: We included all types of publications (e.g., abstract, review articles, original research papers, and editorials) that assessed different doses of heart failure medications based on the search strategy as documented below.

Concepts: We included any type of publications with the terms of selection (as below in the search strategy section) including “high dose,“ “low dose,“ and “heart failure medication.“

Context: We included articles containing data about common medications that can be used for heart failure management, including beta-blockers (BB), angiotensin-converting enzyme inhibitors (ACEi), angiotensin receptor blockers (ARB), mineralocorticoid receptor antagonists (MRA), all types of diuretics, hydralazine with isosorbide dinitrate, ivabradine and angiotensin receptor-neprilysin inhibitor (ARNI) in the context of heart failure management.

### Exclusion criteria

We excluded articles published before 1990, articles that were not in English, or those for which we could not obtain the full text. If we could obtain data about the medication and dose in an English abstract, we included the abstract, even if the full text was written in non-English or was not available. We excluded articles that did not contain at least one category of the medications that we described in the previous section (BB, ACEi, ARB, MRA, diuretics, and ARNI). We excluded articles that did not include information on a low or high dose of such medications. Finally, we excluded articles that investigated medications in the context of other cardiovascular diseases but not heart failure (e.g., hypertension or coronary artery diseases).

### Study selection

**Fig. 1:**
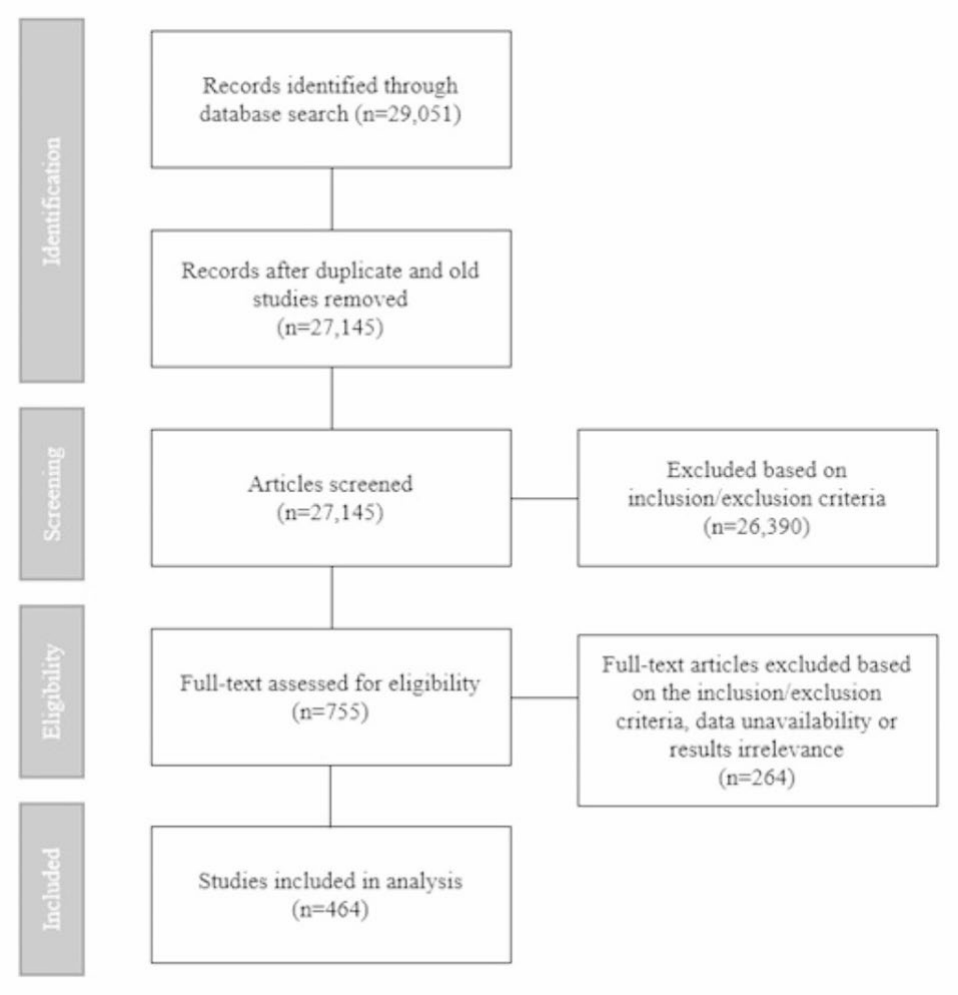
Flow diagram of literature search

The initial search (conducted on 12/18/2020) produced 29,051 articles that were screened for inclusion by one author (MK). The initial screening process produced 4,493 articles (Fig. 1). A second screening produced 755 articles. A second reviewer (CO) reviewed 100 random articles from the initial search list and independently selected articles for inclusion for validation of the inclusion process. Out of 100, there were 24 articles with conflict but mostly resolved by discussion and clarification of the inclusion criteria. This review process was conducted using the web-based program for systematic review, Rayyan [12]. After discussion, 19 more articles were included, and 774 were selected for further review. The final in-depth review further excluded 264 articles resulting in a total of 464 articles for analysis (Fig. 1).

### Data extraction

The initial data extraction was conducted separately by two authors (MK and QW), with The definition of high and low intensity, the medication name, and the characteristics of the study were collected.

## Results

### Number of articles

**Fig. 2:**
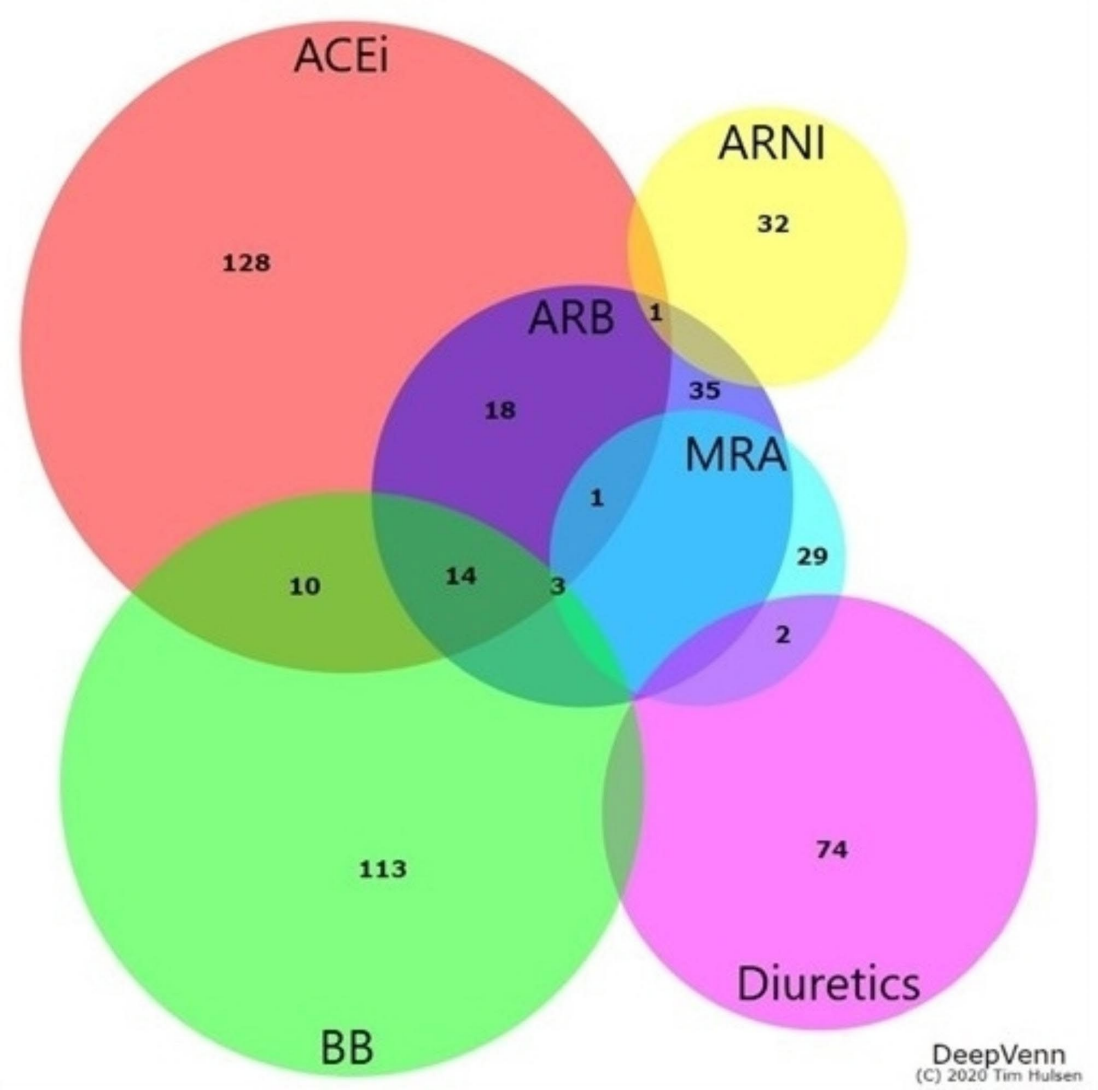
Number of studies for each medication classification (Venn-diagram). ((Venn diagram created by a web application by Hulsen, et al. ACEi: angiotensin converting enzyme inhibitors, ARB: angiotensin receptor blocker, BB: beta-blockers, MRA: Mineralocorticoid receptor antagonists, and ARNI: angiotensin receptor-neprilysin inhibitor. The Venn diagram could not show 2 studies with both ACEi + ARB + diuretics + BB, 1 study with both ACEi + ARB + diuretics + BB + MRA and 1 study with both ACEi + diuretics + BB + MRA))

Out of the total 464 articles, 144 had information about BB, 179 articles about ACEi, 75 articles about ARB, 80 articles about diuretics, 37 articles about MRA, and 33 articles about ARNI, and there were multiple overlaps. Specifically, 14 articles contained information on the dose intensity of BB, ACEi, and ARB, and 18 had information about both ACEi and ARB. Figure 2 shows the Venn diagram of the number of articles with information about each medication and their overlaps [13].

### Characteristics of the articles

The most common study types were prospective studies; among the total articles for each medication classification, 45.1% for BB, 39.7% for ACEi, 36.0% for ARB, 45.0% for diuretics, 35.1% for MRA, and 42.4% for ARNI were prospective studies. For manuscript type, except ARNI (30.3%), the full-text manuscript was more common than abstract; 74.3% for BB, 84.9% for ACEi, 80.0% for ARB, 72.5% for diuretics, and 67.6% for MRA. When assessing how the articles used the definition of high or low intensity, it was most often used to categorize intensity level and evaluate the association between the dose and outcomes; 75.7% for BB, 57.0% for ACEi, 65.3% for ARB, 78.8% for diuretics, 59.5% for MRA and 90.9% for ARNI (Table 1). When assessed if the study identified the rationale of their intensity definition, more than half of the articles identified the sources for BB (52.8%), ACEi (53.1%), or ARB (60.0%), but not for diuretics (37.5%), MRA (43.2%) or ARNI (12.1%). Table 2 shows the countries of the included studies.

**Table 1:** Characteristics of included studies

	BB	ACEi	ARB	Diuretics	MRA	ARNI
	N = 144	N = 179	N = 75	N = 80	N = 37	N = 33
Study Type						
Prospective study	65 (45.1%)	71 (39.7%)	27 (36.0%)	36 (45.0%)	13 (35.1%)	14 (42.4%)
Retrospective study	35 (24.3%)	26 (14.5%)	13 (17.3%)	18 (22.5%)	4 (10.8%)	13 (39.4%)
Review/editorial article	29 (20.1%)	56 (31.3%)	25 (33.3%)	7 (8.8%)	13 (35.1%)	0 (0.0%)
Other type	15 (10.4%)	26 (14.5%)	10 (13.3%)	19 (23.8%)	7 (18.9%)	6 (18.2%)
Manuscript Type						
Full-text manuscript	107 (74.3%)	152 (84.9%)	60 (80.0%)	58 (72.5%)	25 (67.6%)	10 (30.3%)
Abstract	37 (25.7%)	27 (15.1%)	15 (20.0%)	22 (27.5%)	12 (32.4%)	23 (69.7%)
How did they use the definition?						
To categorize intensity level and evaluate the association between the dose and outcomes	109 (75.7%)	102 (57.0%)	49 (65.3%)	63 (78.8%)	22 (59.5%)	30 (90.9%)
To discuss about the association between the dosage and outcome during the discussion or review	32 (22.2%)	69 (38.5%)	24 (32%)	15 (18.8%)	15 (40.5%)	3 (9.1%)
Miscellaneous	3 (2.1%)	8 (4.5%)	2 (2.7%)	2 (2.5%)		
Did they describe the source of literature for the definition used in the literature?						
Not identified or developed their own definition	68 (47.2%)	84 (46.9%)	30 (40.0%)	50 (62.5%)	21 (56.8%)	29 (87.9%)
Identified the source of definition	76 (52.8%)	95 (53.1%)	45 (60.0%)	30 (37.5%)	16 (43.2%)	4 (12.1%)

**Table 2:** Countries of the included studies

	BB	ACEi	ARB	Diuretics	MRA	ARNI
	N = 144	N = 179	N = 75	N = 80	N = 37	N = 33
Country of the study						
U.S.A	44 (30.6%)	56 (31.3%)	24 (32.0%)	15 (18.8%)	13 (35.1%)	3 (9.1%)
Canada	8 (5.6%)	15 (8.4%)	8 (10.7%)	2 (2.5%)	1 (2.7%)	4 (12.1%)
UK	8 (5.6%)	19 (10.6%)	7 (9.3%)	2 (2.5%)	1 (2.7%)	
Italy	5 (3.5%)	11 (6.1%)	3 (4.0%)	7 (8.8%)	2 (5.4%)	4 (12.1%)
Japan	11 (7.6%)	2 (1.1%)	2 (2.7%)	12 (15.0%)	1 (2.7%)	
Australia	6 (4.2%)	11 (6.1%)	3 (4.0%)	3 (3.8%)	3 (8.1%)	2 (6.1%)
Germany	5 (3.5%)	12 (6.7%)	7 (9.3%)	1 (1.3%)	1 (2.7%)	
Spain	6 (4.2%)	3 (1.7%)	3 (4.0%)	2 (2.5%)		7 (21.2%)
Denmark	8 (5.6%)	4 (2.2%)	4 (5.3%)	2 (2.5%)		
Netherlands	4 (2.8%)	4 (2.2%)		7 (8.8%)	1 (2.7%)	
Portugal	1 (0.7%)	4 (2.2%)	3 (4.0%)	5 (6.3%)		2 (6.1%)
Greece		5 (2.8%)		7 (8.8%)		
Sweden	4 (2.8%)	2 (1.1%)	1 (1.3%)	2 (2.5%)	2 (5.4%)	
China	3 (2.1%)	1 (0.6%)	1 (1.3%)	1 (1.3%)	4 (10.8%)	1 (3.0%)
France	1 (0.7%)	5 (2.8%)	1 (1.3%)		2 (5.4%)	2 (6.1%)
Brazil	4 (2.8%)	4 (2.2%)		1 (1.3%)		
South Korea	6 (4.2%)		1 (1.3%)			1 (3.0%)
Switzerland	2 (1.4%)	3 (1.7%)	1 (1.3%)	2 (2.5%)		
Austria	2 (1.4%)	5 (2.8%)				
Singapore	2 (1.4%)	2 (1.1%)	2 (2.7%)		1 (2.7%)	
China Taiwan	3 (2.1%)	1 (0.6%)				2 (6.1%)
Belgium		2 (1.1%)	2 (2.7%)		1 (2.7%)	
Ireland		2 (1.1%)			1 (2.7%)	2 (6.1%)
Turkey	2 (1.4%)			2 (2.5%)		
Israel	1 (0.7%)	1 (0.6%)	1 (1.3%)	1 (1.3%)		
New Zealand	1 (0.7%)	1 (0.6%)		1 (1.3%)		1 (3.0%)
India	3 (2.1%)					
Ukraine	1 (0.7%)			1 (1.3%)	1 (2.7%)	
Norway	1(0.7%)	1(0.6%)				
Czechia		1(0.6%)	1 (1.3%)			
Croatia				1 (1.3%)	1 (2.7%)	
South Africa	1(0.7%)					
Not Identified	1(0.7%)					
Hong Kong		1(0.6%)				
Malaysia		1(0.6%)				
Czech				1 (1.3%)		
Puerto Rico				1 (1.3%)		
Thailand				1 (1.3%)		
Hungary					1 (2.7%)	
Bulgaria						1 (3.0%)
Poland						1 (3.0%)

### Definition of high or low dose

We identified 44 heart failure medications through an in-depth review of the 464 articles, 13 medications for BB, 16 medications for ACEi, 8 medications for ARB, 4 medications for diuretics, 2 medications for MRA, and 1 medication for ARNI (Table 3). We included all potential heart failure medications including non-loop diuretics (metolazone), although non-loop diuretics are not specifically mentioned in heart failure management guidelines [2, 3]. For hydralazine with isosorbide dinitrate or ivabradine, we could not identify any eligible articles with dose intensity and focusing on heart failure management. We converted all doses to total daily doses and assessed the most frequently used definitions of high or low dose for each medication. We could not determine the most frequently used definition of four medications since none of the definitions dominated others (nadolol, pindolol, cilazapril, and torsemide). Finally, we report the most commonly used definition of high and low dose intensity for 40 distinct medications.

### Beta-blockers (BB)

For the BB intensity definition, we reviewed a total of 144 articles (supplemental file 3). Among them, 46 articles specified the intensity using the medication category rather than defining the intensity by an individual medication and dose. Out of the 46 articles, 26 defined the low intensity as < 50% of the maximum recommended target dose and high as ≥ 50% of the target dose. Per the American College of Cardiology/American Heart Association Guideline for the Management of Heart Failure [1], the maximum recommended target doses of BB are 10 mg for bisoprolol, 100 mg for carvedilol, 80 mg for carvedilol CR, and 200 mg for metoprolol succinate. Table 3 shows the most frequently used definitions of low or high doses of these BBs include carvedilol (low < 25 mg and high ≥ 25 mg), carvedilol CR (low < 40 mg and high ≥ 40 mg), metoprolol succinate (low < 100 mg and high ≥ 100 mg) and bisoprolol (low < 5 mg.

and high ≥ 5 mg). Other BBs that were not described in the guidelines but still have been used in managing heart failure were also identified. However, the number of articles is relatively small, including nebivolol (low < 5 mg and high ≥ 5 mg), atenolol (low < 50 mg and high ≥ 50 mg), acebutolol (low < 200 mg and high ≥ 200 mg), propranolol (low < 80 mg and high ≥ 80 mg), sotalol (low < 160 mg and high ≥ 160 mg), timolol (low < 10 mg and high ≥ 10 mg), and labetalol (low < 200 mg and high ≥ 200 mg). Nadolol and pindolol did not show any consensus for dose intensity.

### Angiotensin converting enzyme inhibitors (ACEi)

Among the 179 articles that contained the definition of low or high dose for ACEi (supplemental file 4), 30 used the medication classification when defining the low or high intensity, similar to the case of BB.

**Table 3:** Most commonly used dailty doses to identify low or high dose intensity in the literature

Medication	Number of studies included	Number of studies with most common definition	Most common definition	
			Low	High
Beta-blockers				
Carvedilol	119 studies	40 studies	< 25 mg	≥ 25 mg
Carvedilol CR	47 studies	27 studies	< 40 mg	≥ 40 mg
Metoprolol succinate	95 studies	31 studies	< 100 mg	≥ 100 mg
Bisoprolol	82 studies	30 studies	< 5 mg	≥ 5 mg
Nebivolol	5 studies	2 studies	< 5 mg	≥ 5 mg
Atenolol	7 studies	3 studies	< 100 mg	≥ 100 mg
Acebutolol	2 studies	2 studies	< 200 mg	≥ 200 mg
Propranolol	2 studies	2 studies	< 80 mg	≥ 80 mg
Sotalol	2 studies	2 studies	< 160 mg	≥ 160 mg
Timolol	2 studies	2 studies	< 10 mg	≥ 10 mg
Labetalol	1 study	1 study	< 200 mg	≥ 200 mg
Nadolol	2 studies	No consensus		
Pindolol	2 studies	No consensus		
ACE-inhibitors				
Lisinopril	129 studies	71 studies	≤ 5 mg	≥ 32.5 mg
Enalapril	108 studies	23 studies	< 10 mg	≥ 10 mg
Captopril	68 studies	25 studies	< 75 mg	≥ 75 mg
Perindopril	40 studies	16 studies	< 4 mg	≥ 4 mg
Ramipril	45 studies	17 studies	< 5 mg	≥ 5 mg
Trandolapril	36 studies	14 studies	< 2 mg	≥ 2 mg
Fosinopril	41 studies	13 studies	< 20 mg	≥ 20 mg
Quinapril	40 studies	15 studies	< 20 mg	≥ 20 mg
Moexipril	1 study	1 study	≤ 7.5 mg	> 7.5 mg
Benazepril	9 studies	3 studies	< 10 mg	≥ 10 mg
Imidapril	2 studies	2 studies	≤ 10 mg	> 10 mg
Delapril	1 study	1 study	≤30 mg	> 30 mg
Spirapril	2 studies	2 studies	≤ 6 mg	> 6 mg
Temocapril	1 study	1 study	≤ 2 mg	> 2 mg
Cilazapril	4 studies	No consensus		
ARB				
Losartan	62 studies	38 studies	≤ 50 mg	≥ 150 mg
Candesartan	27 studies	9 studies	< 16 mg	≥ 16 mg
Valsartan	30 studies	11 studies	< 160 mg	≥ 160 mg
Irbesartan	7 studies	2 studies	≤ 150 mg	> 150 mg
Olmesartan	3 studies	2 studies	≤ 10 mg	> 10 mg
Telmisartan	4 studies	2 studies	≤ 40 mg	> 40 mg
Azilsartan	2 studies	2 studies	≤ 80 mg	> 80 mg
Eprosartan	2 studies	2 studies	≤ 400 mg	> 400 mg
Diuretics				
Furosemide	70 studies	12 studies	≤ 80 mg	> 80 mg
Bumetanide	4 studies	2 studies	< 10 mg	≥ 10 mg
Metolazone	1 study	1 study	≤ 5 mg	> 5 mg
Torsemide	4 studies	No consensus		
MRA				
Spironolactone	35 studies	10 studies	≤ 25 mg	> 25 mg
Eplerenone	5 studies	4 studies	< 25 mg	≥ 25 mg
ARNI				
Sacubitril/Valsartan	33 studies	27 studies	48/52 mg	194/206 mg

Among them, 12 articles defined low as < 50% of the maximum target dose and high as ≥ 50% of the maximum target dose. The ACEis in the guideline with the maximum recommended target doses include captopril, enalapril, fosinopril, lisinopril, perindopril, quinapril, ramipril, and trandolapril. Therefore, most of the intensity definition followed this guideline, with low being < 50% of the maximum target and high being ≥ 50% of the maximum target: including enalapril (low < 10 mg and high ≥ 10 mg), captopril (low < 75 mg and high ≥ 75 mg), perindopril (low < 4 mg and high ≥ 4 mg), ramipril (low < 5 mg and high ≥ 5 mg), trandolapril (low < 2 mg and high ≥ 2 mg), fosinopril (low < 20 mg and high ≥ 20 mg) and quinapril (low < 20 mg and high ≥ 20 mg), except lisinopril. The dose intensity for lisinopril was most frequently defined using the definition of the Assessment of Treatment with Lisinopril and Survival (ATLAS) trial [14], low dose lisinopril as 2.5 ~ 5 mg and high dose as 32.5 ~ 35 mg. The remaining ACEis with a very small number of articles included moexipril (low ≤ 7.5 mg and high > 7.5 mg), benazepril (low < 10 mg and high ≥ 10 mg), imidapril (low ≤ 10 mg and high > 10 mg), delapril (low ≤ 30 mg and high > 30 mg), spirapril (low ≤ 6 mg and high > 6 mg) and temocapril (low ≤ 2 mg and high > 2 mg). Cilazapril was mentioned in 4 articles, but there was no consensus on the dose intensity definition.

### Angiotensin receptor blockers (ARB)

For ARB, we identified 75 articles (supplemental file 5). Among them, 14 articles used the “medication classification” when defining the intensity rather than using the name of an individual medication, and 7 of them defined low dose as < 50% of the maximum target dose and high dose as ≥ 50% of the maximum target dose. The guideline for managing heart failure recommends a maximum target dose of the following three ARBs: candesartan, losartan, and valsartan. Among them, the dose intensities for candesartan and valsartan were most frequently defined by such definition, low being < 16 mg and high being ≥ 16 mg for candesartan and low being < 160 mg and high being ≥ 160 mg for valsartan. The dose intensity for losartan was most frequently defined based on the dose used in the Heart failure Endpoint evaluation of Angiotensin II Antagonist Losartan (HEAAL) trial, with a definition of low ≤ 50 mg and high ≥ 150 mg [15]. Other ARBs and their intensity definitions that we identified include irbesartan (low dose ≤ 150 mg and high dose > 150 mg), olmesartan (low dose ≤ 10 mg and high dose > 10 mg), telmisartan (low dose ≤ 40 mg and high dose > 10 mg), azilsartan (low does ≤ 80 mg and high dose > 80 mg), and eprosartan (low dose ≤ 400 mg and high dose > 400 mg).

### Diuretics

Among the 80 articles that addressed diuretics, furosemide appeared most frequently in 70 articles (supplemental file 6). The most frequently used definition of a low dose for furosemide was < 80 mg, and a high dose ≥ 80 mg, as defined in 12 articles. Other diuretics that we were able to find the consensus in definitions included bumetanide (low dose < 10 mg and high dose ≥ 10 mg) and metolazone (low dose ≤ 5 mg and high dose > 5 mg). Four articles mentioned torsemide, but we could not find a consensus on the definition of dose intensity among them.

### Mineralocorticoid receptor antagonists (MRA)

For MRAs, among the total 37 articles (supplemental file 7), 35 addressed spironolactone, and 5 mentioned eplerenone. The most frequently used definition of low spironolactone dose was ≤ 25 mg, and the high dose was > 25 mg. The high dose of eplerenone was defined as a low dose of < 25 mg and a high dose of ≥ 25 mg.

### Angiotensin receptor-neprilysin inhibitor (ARNI)

We found 33 articles mentioning ARNI, which includes only one medication, sacubitril/valsartan, and the common definition of the low dose was 48/52 mg a day. The high dose was 194/206 mg daily (supplemental file 8).

## Discussion

**Fig. 3:**
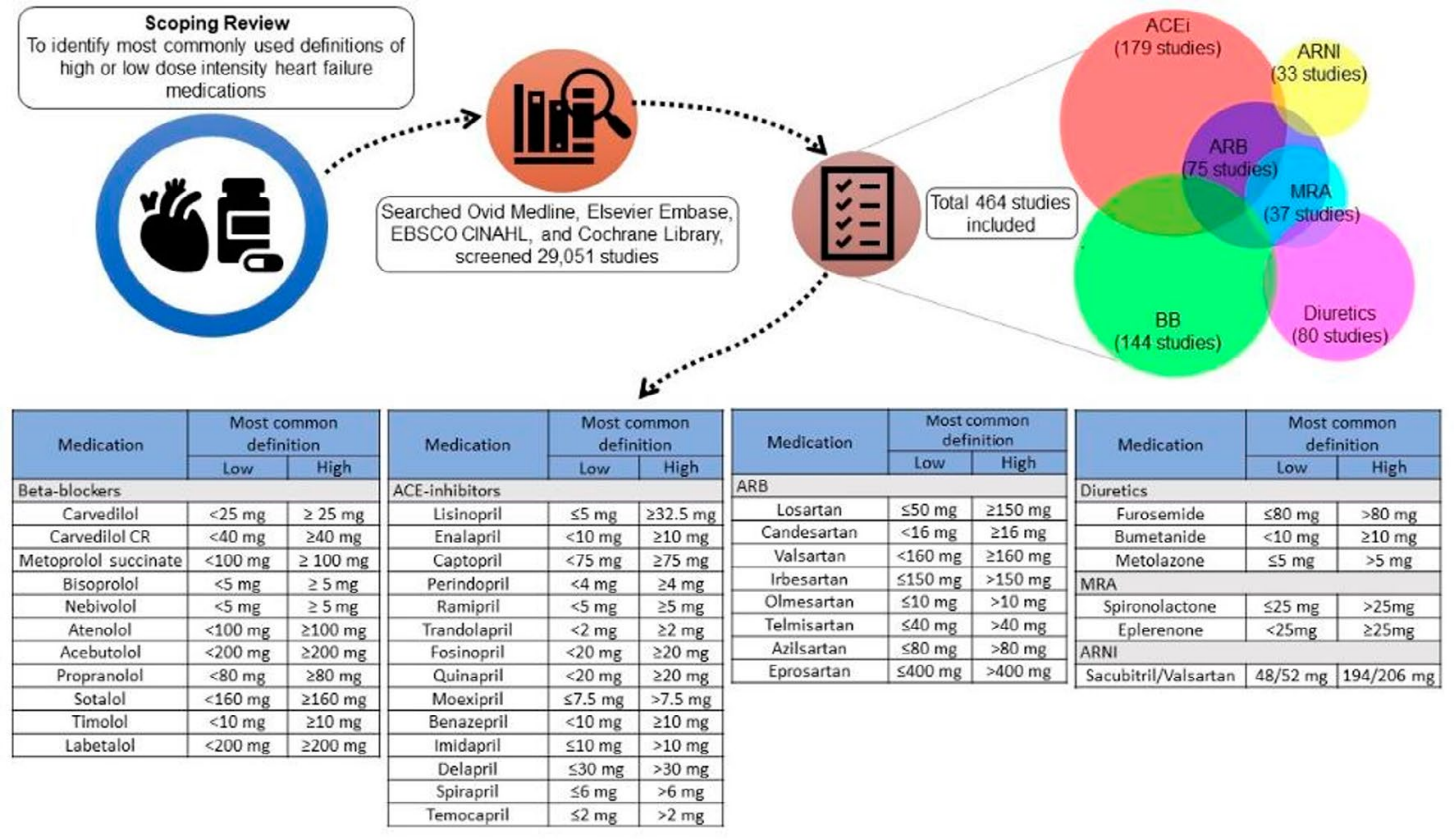
Graphical summary of the scoping review and results. (ACEi: angiotensin-converting enzyme inhibitors, ARB: angiotensin receptor blockers, ARNI: angiotensin receptor-neprilysin inhibitor, BB: beta-blocker, MRA: mineralocorticoid receptor antagonists)

The current scoping review article identfieid the most frequently used definitions for low or high dose intensity for medications used to manage heart failure. Through an extensive and comprehensive review, we identified the most frequently used definitions for most medications used for heart failure management (Fig. 3), except for nadolol, pindolol, cilazapril, and torsemide.

To our knowledge, our study is the first review that establishes the definitions for low or high-dose intensity dosing for most potential medications that can be used for heart failure management. Among the articles for BBs, the most frequently used cut-off to identify high or low doses was 50% of the maximum dose recommended by the guideline for heart failure management. However, we could not find a scientific rationale for this definition. Similarly, other articles used the percentage of the target dose as an indicator to differentiate the intensity (0–25% of the target dose as a low dose or 75% of the target dose as a high dose) [16, 17]. However, for other medications, we identified a pattern that if there is a pivotal clinical trial of a certain medication with a pre-determined definition of high or low dose, that definition dominates others. For example, for ACEi, most studies mentioned lisinopril, and more than half of the studies used the ATLAS trial’s definition of dose intensity [14]. We found a similar pattern for ARBs, with losartan being the most commonly mentioned medication for dose intensity, and most studies using the HEAAL trial for the definition of dose intensity [15]. For diuretics or MRA, such as furosemide or spironolactone, there was no distinctive trial or study that was most frequently used to define intensity. For spironolactone, the Aldosterone Targeted Neurohormonal Combined with Natriuresis Therapy in Heart Failure (ATHENA-HF) trial defined the low dose of spironolactone as 25 mg and the high dose as 100 mg, but this definition was not the most common [18]. However, low doses of 25 mg and less were used most frequently, along with high doses of more than 25 mg (not 100 mg). For ARNI, there was only one medication – sacubitril/valsartan with a predominant dose definition as low being 48/52 mg and high being 194/206 mg.

These results could be valuable in clinical settings to guide the management of heart failure and more easily assess the intensity of heart failure treatment. In heart failure management, the guideline recommends reaching the maximum target dose of each medication for clinical benefit. However, there is still a wide gap between the target doses and real-world doses. For example, the Cardiac Insufficiency Bisoprolol Study in Elderly (CIBIS-ELD) trial assessed the doses of bisoprolol and carvedilol in older patients with heart failure, and only 55% of the cohort reached at least 50% of the target dose [19]. Although the reasons for the gap between guidelines and real-world treatment are not yet clear, establishing a threshold for dose intensity is an important step to understanding this discrepancy. The results of the current study could be a stepping stone to establish such new practical threshold.

In addition, whether a patient is able to tolerate high dose therapy could be an important marker for beneficial outcomes and adverse events. For example, with ACEi, the ATLAS trial showed no difference in mortality between the low and high-dose groups, but the high-dose group had a lower hospitalization rate for heart failure [14]. However, the high-dose group experienced a higher rate of dizziness and renal insufficiency. For clinicians, being mindful of which intensity the patient is on for heart failure management will assist in clinical practice and in the decision making process.

Furthermore, the concept of polypill in cardiovascular diseases has been receiving attention in response to the concern of medication-related problems from polypharmacy and noncompliance [20]. A polypill is a single pill containing multiple generic medications and was developed to increase compliance among patients, especially those who are supposed to take multiple medications. Therefore, patients with heart failure, especially older adults with polypharmacy, can benefit from the polypill strategy. Furthermore, the polypill strategy in heart failure management focuses on using low doses, acknowledging that older adults and physicians are less likely to prescribe multiple medications due to fear of medication-related problems with high doses. The results from our study provide fundamental information regarding the dose intensity in developing multiple polypill combinations.

Although the current study has the strength that it is from a comprehensive scoping review with the potential to be used in practice and research, the authors also acknowledge several limitations. First, since the study is a scoping review, the results map the existing literature with a large sample of heterogenous articles, including all types of articles (e.g., abstract, review articles, original research papers, and editorials). Therefore, unlike a systematic review, the study could not produce an in-depth evidence synthesis. Furthermore, most of the studies did not distinguish between heart failure with reduced ejection fraction and heart failure with preserved ejection fraction, although their management could be quite different. A more detailed critical appraisal and evaluation of the level of evidence for dose intensity for each individual medication depending on the ejection fraction would require a systematic review and meta-analysis; however, this may not be practical given the significant number of heart failure therapies and the possible exclusion of useful therapies that lack adequate evidence for meta-analysis for dose intensity. Second, although the authors extensively searched for all the potential articles defining the high or low dose intensity, the search term high-dose intensity or low-dose intensity could have excluded several articles. However, the authors used search terms with maximal possible combinations of the terms in four different search engines (supplemental file 8). Therefore, we do not believe that we had many articles excluded because of an exclusive search term. Third, the current study chose heart failure medications based on the heart failure management guideline published in 2017 [1], and the search was conducted in 2020. Therefore, our research did not include novel heart medications such as sodium-glucose cotransporter (SGLT) -2 inhibitors or Glucagon-Like Peptide 1 Receptor Agonists. For example, SGLT-2 inhibitors are included in the heart failure management guideline in 2022 [21]. SGLT-1/2 inhibitor was approved for heart failure management by the U.S. Food and Drug Administration in 2023 [22]. Of course, given that more medications are added to the standard heart failure management, future research including novel heart failure medications is also warranted.

## Conclusion

In conclusion, through an extensive scoping review, the authors present the most frequently used definition of low and high doses of 40 heart failure medications. The definitions proposed for low and high intensity dose will help guide clinical practice and future articles evaluating the effects of dose intensity on key clinical outcomes.

### Electronic supplementary material

Below is the link to the electronic supplementary material.

Supplementary Material 1

Supplementary Material 2

Supplementary Material 3

Supplementary Material 4

Supplementary Material 5

Supplementary Material 6

Supplementary Material 7

Supplementary Material 8

## Data Availability

The data analyzed during the current study are included in the supplemental file.

## List of abbreviations

ACEi: angiotensin-converting enzyme inhibitors
ARB: angiotensin receptor blockers
ARNI: angiotensin receptor-neprilysin inhibitor
ATHENA-HF: Aldosterone Targeted Neurohormonal Combined with Natriuresis Therapy in Heart Failure
ATLAS: Assessment of Treatment with Lisinopril and Survival
BB: beta-blocker
CIBIS-ELD: Cardiac Insufficiency Bisoprolol Study in Elderly
HEAAL: Heart failure Endpoint evaluation of Angiotensin II Antagonist Losartan
MRA: mineralocorticoid receptor antagonists
SGLT: sodium-glucose cotransporter
