# Supplementary material for: How do we define high and low dose intensity of heart failure medications: a scoping review
Source: BMC Cardiovasc Disord. 2023 Sep 27;23:478. doi: 10.1186/s12872-023-03514-2 (PMC10523693; doi:10.1186/s12872-023-03514-2)
Supplement: Supplementary file 1 — Supplementary Material 1 [file 12872_2023_3514_MOESM1_ESM.docx]

**Supplemental file 1: Preferred Reporting Items for Systematic reviews and Meta-Analyses extension for Scoping Reviews (PRISMA-ScR) Checklist**

for

**How do we define high and low dose intensity of heart failure medications: a scoping review**

Min Ji Kwak^a^, Qian Wang^b^, Chukwuma Onyebeke^c^, Travis Holder^d^, Parag Goyal^c^, Rajender Aparasu^e^, Abhjeet Dhoble^f^, Holly M. Holmes^a^

a. Division of Geriatric and Palliative Medicine, McGovern Medical School, The University of Texas Health Science Center at Houston

b. Department of Biostatistics, School of Public Health, The University of Texas Health Science Center at Houston

c. Department of Medicine, Weill Cornell Medicine

d. Houston Academy of Medicine-Texas Medical Center Library, Texas Medical Center

e. Department of Pharmaceutical Health Outcomes and Policy, University of Houston

f. Division of Cardiovascular Medicine, The University of Texas Health Science Center at Houston

Table 1-1: Preferred Reporting Items for Systematic reviews and Meta-Analyses extension for Scoping Reviews (PRISMA-ScR) Checklist

**Supplemental table 1-1: Preferred Reporting Items for Systematic reviews and Meta-Analyses extension for Scoping Reviews (PRISMA-ScR) Checklist**

| **SECTION** | **ITEM** | **PRISMA-ScR CHECKLIST ITEM** | **REPORTED AT:** |
| --- | --- | --- | --- |
| **TITLE** | | | |
| Title | 1 | Identify the report as a scoping review. | Title page |
| **ABSTRACT** | | | |
| Structured summary | 2 | Provide a structured summary that includes (as applicable): background, objectives, eligibility criteria, sources of evidence, charting methods, results, and  conclusions that relate to the review questions and objectives. | Abstract page |
| **INTRODUCTION** | | | |
| Rationale | 3 | Describe the rationale for the review in the context of what is already known. Explain why the review  questions/objectives lend themselves to a scoping review approach. | Background 2^nd^ paragraph |
| Objectives | 4 | Provide an explicit statement of the questions and objectives being addressed with reference to their key elements (e.g., population or participants, concepts, and context) or other relevant key elements used to conceptualize the review questions and/or objectives. | Background 2^nd^ paragraph |
| **METHODS** | | | |
| Protocol and registration | 5 | Indicate whether a review protocol exists; state if and where it can be accessed (e.g., a Web address); and if available, provide registration information, including the registration number. | Methods 2^nd^ paragraph, under “Search strategy” |
| Eligibility criteria | 6 | Specify characteristics of the sources of evidence used as eligibility criteria (e.g., years considered, language, and publication status), and provide a rationale. | Methods 3rd paragraph, under “Inclusion criteria” |
| Information sources | 7 | Describe all information sources in the search (e.g., databases with dates of coverage and contact with authors to identify additional sources), as well as the date the most recent search was executed. | Methods 2^nd^ paragraph, under “Search strategy”  Supplemental file 2 |
| Search | 8 | Present the full electronic search strategy for at least 1  database, including any limits used, such that it could be repeated. | Methods 2^nd^ paragraph, under “Search strategy”  Supplemental file 2 |
| Selection of sources of evidence | 9 | State the process for selecting sources of evidence (i.e., screening and eligibility) included in the scoping review. | Methods 5^th^ paragraph, under “Study selection” |
| Data charting process | 10 | Describe the methods of charting data from the included sources of evidence (e.g., calibrated forms or forms that have been tested by the team before their use, and whether data charting was done independently or in duplicate) and any processes for obtaining and confirming data from investigators. | Methods 5^th^ and 6^th^ paragraph, under “Study selection” and “Data extraction” |
| Data items | 11 | List and define all variables for which data were sought and any assumptions and simplifications made. | Methods 6^th^ paragraph, under “Data extraction” |
| Critical appraisal of individual sources of evidence | 12 | If done, provide a rationale for conducting a critical appraisal of included sources of evidence; describe the  methods used and how this information was used in any data synthesis (if appropriate). | Not done, but described as a limitation at Discussion 6^th^ paragraph. |
| Synthesis of results | 13 | Describe the methods of handling and summarizing the data that were charted. | Methods 5^th^ and 6^th^ paragraph, under “Study selection” and “Data extraction” |
| **RESULTS** | | | |
| Selection of sources of evidence | 14 | Give numbers of sources of evidence screened, assessed for eligibility, and included in the review, with reasons for exclusions at each stage, ideally using a flow diagram. | Figure 1 |
| Characteristics of sources of evidence | 15 | For each source of evidence, present characteristics for which data were charted and provide the citations. | Table 1 and 2. Supplemental files (3-8) |
| Critical appraisal within sources of evidence | 16 | If done, present data on critical appraisal of included sources of evidence (see item 12). | Not done, but described as a limitation at Discussion 6^th^ paragraph. |
| Results of individual sources of evidence | 17 | For each included source of evidence, present the relevant data that were charted that relate to the review questions and objectives. | Table 1,2 and 3, and Supplemental files (3-8) |
| Synthesis of results | 18 | Summarize and/or present the charting results as they relate to the review questions and objectives. | Table 3 |
| **DISCUSSION** | | | |
| Summary of evidence | 19 | Summarize the main results (including an overview of concepts, themes, and types of evidence available), link to the review questions and objectives, and consider the relevance to key groups. | Discussion 1^st^ paragraph |
| Limitations | 20 | Discuss the limitations of the scoping review process. | Discussion 6^th^ paragraph |
| Conclusions | 21 | Provide a general interpretation of the results with respect to the review questions and objectives, as well  as potential implications and/or next steps. | Discussion 2^nd^ and 3^rd^, and 4^th^ paragraphs |
| **FUNDING** | | | |
| Funding | 22 | Describe sources of funding for the included sources of evidence, as well as sources of funding for the scoping review. Describe the role of the funders of the scoping review. | Declaration 5^th^ paragraph, under “Funding” |

*Check List From:* Tricco AC, Lillie E, Zarin W, O'Brien KK, Colquhoun H, Levac D, et al. PRISMA Extension for Scoping Reviews (PRISMAScR): Checklist and Explanation. Ann Intern Med. 2018;169:467–473. [doi: 10.7326/M18-0850.](http://annals.org/aim/fullarticle/2700389/prisma-extension-scoping-reviews-prisma-scr-checklist-explanation)
