# Supplementary material for: How do we define high and low dose intensity of heart failure medications: a scoping review
Source: BMC Cardiovasc Disord. 2023 Sep 27;23:478. doi: 10.1186/s12872-023-03514-2 (PMC10523693; doi:10.1186/s12872-023-03514-2)
Supplement: Supplementary file 2 — Supplementary Material 2 [file 12872_2023_3514_MOESM2_ESM.docx]

**Supplemental file 2: Search Terms**

**for**

**How do we define high and low dose intensity of heart failure medications: a scoping review**

Min Ji Kwak^a^, Qian Wang^b^, Chukwuma Onyebeke^c^, Travis Holder^d^, Parag Goyal^c^, Rajender Aparasu^e^, Abhjeet Dhoble^f^, Holly M. Holmes^a^

a. Division of Geriatric and Palliative Medicine, McGovern Medical School, The University of Texas Health Science Center at Houston

b. Department of Biostatistics, School of Public Health, The University of Texas Health Science Center at Houston

c. Department of Medicine, Weill Cornell Medicine

d. Houston Academy of Medicine-Texas Medical Center Library, Texas Medical Center

e. Department of Pharmaceutical Health Outcomes and Policy, University of Houston

f. Division of Cardiovascular Medicine, The University of Texas Health Science Center at Houston

Table 2-1: Search Terms

**Supplemental table 2-1: Search Terms (searched on 12/18/2020)**

1. MEDLINE (Ovid)

#1. heart failure/ or heart failure, systolic/ or ((cardiac or heart or myocardial) adj2 (decompensation* or de-compensation* or dysfunction* or failure*)).mp. or cardiomyopath*.mp.

#2. ((drug* or medica* or pharmac*) adj2 (manag* or therap*)).mp. or Drug Therapy/

#3. ((high* or low* or increas* or decreas*) adj2 (dos* or intens*)).tw,kw. or Drug Dosage Calculations/ or Dose-Response Relationship, Drug/

#4. 1 and 2 and 3

2. EMBASE

#1. 'drug therapy'/de OR 'diuretic therapy'/de OR 'combination drug therapy'/exp OR 'dosage schedule comparison'/de OR 'dose densification'/de OR 'drug choice'/de OR 'drug dose comparison'/de OR 'drug dose escalation'/de OR 'drug dose increase'/de OR 'drug dose intensification'/de OR 'drug dose reduction'/de OR 'drug dose regimen'/de OR 'drug dose sequence'/de OR 'drug dose titration'/de OR 'drug intermittent therapy'/de OR 'drug megadose'/de OR 'drug microdose'/de OR 'drug mixture'/de OR 'drug pulse therapy'/de OR 'drug repositioning'/de OR 'drug substitution'/de OR 'inappropriate prescribing'/de OR 'low drug dose'/de OR 'maintenance drug dose'/de OR 'multiple drug dose'/de OR 'optimal drug dose'/de OR 'pharmaceutical care'/de OR 'polypharmacy'/de OR 'recommended drug dose'/de OR (((drug* OR medica* OR pharmac*) NEAR/2 (manag* OR therap*)):ab,kw,ti)

#2. 'heart failure'/de OR 'systolic dysfunction'/exp OR ((cardiac OR heart OR myocardial) NEAR/3 (decompensat* OR 'de compensat*' OR failure*))

#3. 'drug dose'/exp OR 'dose response'/exp OR ((high* OR low* OR increas* OR decreas*) NEAR/2 (dos* OR intens*))

#4. #1 AND #2 AND #3

3. CINAHL

S1 ((MH "Drug Therapy") OR (MH "Drug Therapy, Combination+") OR (MH "Inappropriate Prescribing") OR (MH "Polypharmacy")) OR ((drug* OR medica* OR pharmac*) N2 (manag* OR therap*))

S2 (MH "Heart Failure+") OR ((cardiac OR heart OR myocardial) n3 (decompensat* or de-compensat* OR failure*))

S3 (MH "Dose-Response Relationship") OR ((decreas* OR high* OR increas* OR low*) N2 (dos* OR intens*))

S4 S1 AND S2 AND S3

4. Cochrane

#1: MeSH descriptor: [Heart Failure] this term only

#2: MeSH descriptor: [Heart Failure, Systolic] this term only

#3: ((cardiac or heart or myocardial) NEAR/2 (decomp* or de-comp* or dysfunction* or fail* OR insuff*))

#4: cardiomyopath*

#5: #1 OR #2 OR #3 OR #4

#6: ((drug* or medica* or pharmac*) NEAR/2 (manag* or therap*))

#7: MeSH descriptor: [Drug Dosage Calculations] this term only

#8: MeSH descriptor: [Dose-Response Relationship, Drug] this term only

#9: ((high* OR low* OR increas* OR decreas*) NEAR/2 (dos* or intens*))

#10: #7 OR #8 OR #9

#11: #5 AND #6 AND #10
